# A Case Report of Favourable Response of Polymyositis to Methotrexate Monotherapy

**DOI:** 10.31138/mjr.32.1.86

**Published:** 2021-02-08

**Authors:** Dimitrios Daoussis, Ioannis Antonopoulos, Andrew P. Andonopoulos

**Affiliations:** 1 Department of Rheumatology, Patras University Hospital, University of Patras Medical School, Patras, Greece; 2 Rheumatologist, Private practice, Patras, Greece; 3 Emeritus Professor, University of Patras Medical School, Patras, Greece

**Keywords:** Methotrexate, polymyositis, monotherapy, treatment

## INTRODUCTION

Steroids constitute either the main treatment for some rheumatic diseases such as temporal arteritis or may be used as a bridging therapy in others, such as rheumatoid arthritis.^1,2^ The efficacy of steroids in rheumatic diseases is well documented but there was always a concern regarding their well-known side effects.

Polymyositis is generally considered a severe rheumatic disease, and steroids are always used in high doses either as monotherapy or in combination with immunosuppressant agents. One of the most widely used drugs for polymyositis is methotrexate (MTX); other therapeutic options include azathioprine, mycophenolate mofetil, cyclosporine, or biologics such as rituximab.^3,4^ We report herein a rare case of polymyositis that responded favourably to MTX monotherapy without the use of steroids.

## CASE REPORT

Our patient was a 78-year-old retired surgeon who presented with proximal muscle weakness and Raynaud’s phenomenon. The patient was initially seen by rheumatology colleagues in Switzerland, the country in which the patient had been practicing medicine and living. At that time, he complained of difficulty in climbing stairs, getting up from the sitting or lying position and raising his trousers. A creatinine phosphokinase (CPK) measurement showed a value of more than 700u/L, and proximal muscle weakness was reported. An electromyogram revealed a myopathic pattern, whereas a muscle biopsy was reported compatible with polymyositis. Treatment with prednisolone 100mg/d was initiated for ten days with a rapid tapering after that, according to instructions, with prompt normalisation of serum muscle enzymes, but not of muscle weakness. Three weeks following the initiation of steroids, he experienced a severe infection in the form of spondylodiscitis and was hospitalized. Steroids were gradually but swiftly discontinued (the patient was exposed to about a month of steroid treatment in total) and intravenous antibiotics were administered for 4 months, along with laminectomy and drainage of a paraspinal abscess. Following these, the patient gradually recovered. He was off steroids for 10 months but had moderate central muscle weakness for which he presented to us after returning to his home country. The patient had difficulty in rising from the lying position and deep seat whereas he was walking with the help of a cane. Initial laboratory workup revealed a CPK of 851u/L (normal up to 190u/l), a positive rheumatoid factor and antinuclear antibodies (1:320 speckled), positive anti-Ro, and normal thyroid function. A detailed investigation for underlying malignancy was negative. An electromyogram of the proximal muscles was compatible with polymyositis. A diagnosis of polymyositis was made and the need for treatment with steroids was emphasised to the patient. However, he refused this kind of therapy arguing that steroids were responsible for the severe spinal infection he had suffered in the near past. Understanding that we had no other choice, we decided to start him on MTX alone at a dosage of 10mg/ wk, explaining to the patient that this is not an accepted approach for the management of his disease and was advised about the need of close follow-up. At his next appointment, one month later, he reported some improvement of arising from the sitting position. However, decreased quadriceps strength was present as before. His CPK had come down to 667u/L. MTX was increased to 15mg and two months later, the patient claimed a remarkable improvement, especially regarding arising from sitting and lying position, whereas the difficulty climbing stairs was much less. CPK was down to 462u/L. He was continued on methotrexate 15mg per week and two months later he reported a continuous slow and steady improvement. Specifically, he had abandoned the cane whereas arising was not a problem anymore. Serum CPK was 301u/L. At his next appointment, after two months, his improved condition was stable and CPK was 304u/L. He was then lost from follow-up, and we were informed by his relatives that he had died, following a fall while descending the stairs of his house, which caused a lethal cerebral damage.

The present report illustrates the case of an elderly patient with polymyositis treated with MTX alone because he refused to receive steroids. The response of the disease to this therapy was favourable, since a slow, gradual improvement was observed both clinically and biochemically. Unfortunately, our observation of the case was abruptly interrupted by the sudden death of the patient due to an accident. Should this have not happened, we could have more information as to the long-term behaviour of the disease under this regimen. A potential limitation in this case is the lack of a repeat biopsy that was not performed because the patient declined. However, the patient had muscle weakness, elevated CPK and an electromyogram compatible with myositis; all these pointed to the direction of polymyositis relapse. The fact that the patient had been off steroids for 10 months and had raised CPK excludes the possibility of steroid induced myopathy. We performed an electronic search (Medline, Scopus) using the key words “methotrexate monotherapy” and “polymyositis”, and we were unable to identify other cases where MTX was used as monotherapy without steroids. Even though one can say that there is never an absolute contraindication to the use of steroids, there may be rare cases where the use of steroids may be extremely problematic. In such cases, methotrexate alone, under close clinical and laboratory monitoring, might be an acceptable alternative for polymyositis management.
